# Integrating multi-omics approaches to shape legume root system architecture under drought stress: a comprehensive review

**DOI:** 10.3389/fpls.2026.1783318

**Published:** 2026-05-08

**Authors:** Sajad Majeed Zargar, Tamana Khan, Rakeeb Ahmad Mir, Aaqif Zaffar, Uneeb Urwat, Majid Rashid, Parvaze A. Sofi, Baseerat Afroz, Priyanka Deveshwar, Randeep Rakwal, Sajad Ali

**Affiliations:** 1 Proteomics Laboratory, Division of Plant Biotechnology, Sher-e-Kashmir University of Agricultural Sciences & Technology of Kashmir, Srinagar, India; 2 Division of Vegetable Sciences, Sher-e-Kashmir University of Agricultural Sciences & Technology of Kashmir, Srinagar, India; 3 Department of Biotechnology, School of Life Sciences, Central University of Kashmir, Ganderbal, India; 4 Division of Plant Breeding and Genetics, Sher-e-Kashmir University of Agricultural Sciences & Technology of Kashmir, Srinagar, India; 5 Sri Aurobindo College, University of Delhi, Delhi, India; 6 Institute of Health and Sport Sciences, University of Tsukuba, Tsukuba, Japan; 7 Department of Biological Sciences, College of Science, King Faisal University, Al-Ahsa, Hofuf, Saudi Arabia

**Keywords:** abiotic stressors, drought stress, legumes, multi-omics, nutritional security, root system architecture

## Abstract

Drought stress profoundly impacts agricultural productivity, significantly reducing crop yields and global food insecurity. Consequently, improving crops to develop resistance against drought stress is imperative. To combat the adverse impact of climate change on crop productivity, designing the root system architecture (RSA) of crops can be a viable option. RSA is essential for crop adaptation and productivity because most soils have different resource distributions making the spatial root distribution a crucial factor for judicious resource exploitation. RSA involves several structural features like root length, branching angle, and thickness which play key roles in developing crops with desirable roots. Legumes are protein-rich foods and the diverse number of cultivated species makes them one of the most widespread crops. However, legumes are greatly affected by various abiotic stresses like drought and mineral stress. Therefore, it is imperative to understand the environmentally adaptive root development to improve agronomic traits in legumes by employing the OMICS approaches. Several abiotic stressors like drought stress demand proliferative and deep root systems, hence it is important to comprehend the response of RSA to stressors. Further, the genetic regulation (genomics) accompanied by other omics approaches aid in deciphering the biology behind RSA in legumes. The current appraisal may help in devising strategies to modulate legume RSA for efficient uptake of water and nutrients under drought stress.

## 1 Introduction

On global level the efforts to enhance the crop production has significantly hampered by multiple abiotic stresses, leading to a 50–70% drop in crop yields (Francini and Sebastiani, 2019). Even though driven by “Agri-Tech Revolution,” global crop production has reached to maximum of 9.5 billion tons by the mid-2020s, almost a rise of 54% increase since the turn of the century (FAO, 2025). The impacts of climate change and its unpredictability, coupled with arable land degradation, have disrupted plant development and physiology (Fahad et al., 2017). With the expansion of the human population, combined with decreasing soil fertility, drought conditions, and environmental degradation, there is an urgent need to develop crops with better root systems and increased stress tolerance (Lynch, 2022; Zhao et al., 2025). Among various abiotic stresses, drought is the primary factor affecting crop yields by disrupting their physiological processes, growth, and reproductive capacity (Fahad et al., 2017; Lamaoui et al., 2018; Rahim et al., 2025). Plant roots, as the key organ responsible for resource extraction from soil under different stress conditions, need thorough investigation and analysis to understand the mechanisms of stress tolerance. RSA reflects the physical arrangement of roots in space and time and is critically important for soil resource acquisition, which indirectly influences the sustainability of crop systems. Understanding root development patterns and growth dynamics is essential for unraveling the complexity of root architecture and its response mechanisms to environmental challenges (Urfan et al., 2022).

Legumes are essential for food security and environmental health, often grown as intercrops with cereals to boost land productivity (Graham and Vance, 2003). Although legumes can fix nitrogen, there is still a need for more efficient nutrient use to maintain and improve optimal yields. Likewise, utilizing other critical soil resources, especially under stress conditions, is vital for sustaining crop productivity. The ability of root systems to access deep soil layers depends on various architectural, physiological, and anatomical traits described by the “steep, cheap, and deep” ideotype of roots (Lynch, 2013). ‘Steep’ refers to root architectural traits that promote deep rooting, such as growth angles, while ‘cheap’ relates to root system traits that lower the metabolic costs of exploring soil. Because nutrients and water are unevenly distributed in the soil, and in response to multiple abiotic stresses, plants adapt their RSA to optimize resource acquisition from the soil (Xu et al., 2022).

Root hairs are essential for nutrient absorption and penetration of hard soil, and their genetic variation in density and number has been studied in various crop species across low- and high-input systems (Lynch, 2019). Intermediate root architectural traits can access both topsoil and subsoil while lowering the metabolic cost of maintaining multiple root axes (Lynch, 2022). These phenotypes have demonstrated the best yield under conditions of low fertility and drought stress (Strock et al., 2019). Marker-assisted selection (MAS) and genomic selection tools have significantly allowed the precise selection of elite genotypes with multiple stress tolerances (Valliyodan et al., 2017). This can be achieved through various systemic omics approaches, especially advanced genomic analysis and high-throughput genomic platforms (Yaqoob et al., 2023; Mwadzingeni et al., 2017). These methods have been extensively used to identify the genetic signatures of complex quantitative traits, particularly variations in root systems, with precision (Naz et al., 2014). Recently, advanced sensing phenomics techniques have enabled the genetic dissection of several morphological and physiological traits across different environments (Wasaya et al., 2018). Similarly, advanced molecular phenotyping methods like transcriptomics, proteomics, metabolomics, and ionomics have been used to understand trait inheritance at the physiological level. Unlike traditional phenotyping techniques relying on end point physical characteristics, the omics approaches provide deep insights into functional maps of physiological processes to govern the complex traits (Mwadzingeni et al., 2017). The omics approaches aid in quantifying the immediate biological responses at multiple levels such as, from mRNA expression to protein abundance and measuring the fluctuations of small metabolites and minerals to dissect the regulatory network during the phenotypic plasticity of crop plants (Salgotra and Stewart, 2022). Additionally, systems biology approaches are effective in precise identification of molecular signatures pivotal for resilience to stress and resource use efficiency (Jones, 2026). This will allow unravelling of the wide range of functional biomarkers that are less susceptible to environmental challenges rather than relying on field-based observations. Consequently, the generation of multi-dimensional datasets are evolving as indispensable for speeding up modern breeding programs to deeply understand the mechanism necessary to enhance and predict trait performance under diverse and challenging environmental conditions.

Some non-invasive phenotyping platforms now replace traditional methods by evaluating plant stress fitness at the whole-plant and cellular levels (Burridge et al., 2019). The non-invasive phenotyping techniques such as, LemnaTec Scanalyzer (PhenoAIxpert), is versatile high-throughput conveyor system that moves crop plants through highlight specialized imaging cabinets (Shi et al., 2025). This technique uses RGB, 3D Laser Scanning, and Hyperspectral sensors to measure the leaf area, biomass and volumes of crop plants. Also, it is used to investigate the genetic basis if growth dynamics and plant height in crops such as, wheat and maize (Shi et al., 2025). The PlantScreen™ (Photon Systems Instruments) is another non-invasive technique which involves modular robotic systems that integrate the multiple sensors into light -insulated cabinets (Shi et al., 2025; Gill et al., 2022). This technique excels at evaluating chlorophyll fluorescence imaging to map the efficacy of photosynthesis across the whole canopy. This technique also involves thermal imaging to monitor the stomatal conductance and canopy temperature. Scientists also employ Non-invasive Micro-test Technology (NMT) which uses selective microelectrodes to detect the transmembrane transport of ions and small molecules in livings cells and tissues (Bao et al., 2025). This technique measures ion flux at leaf epidermis and root surfaces to evaluate the cellular homeostasis during heavy metal and salinity stress (Bao et al., 2025). Additionally Magnetic Resonance Imaging (MRI) & Positron Emission Tomography (PET) analyzing internal structures and transport mechanism by studying 3D root soil interaction and water content mapping (Mehra et al., 2025).

Although current information about RSA in legumes highlights the importance of using superior phenotypes in crop breeding programs (Afonso et al., 2025; Rascher et al., 2011), significant knowledge gaps remain (Lynch, 2022). It is also essential to understand root phenotypic fitness in response to changing agroecological conditions. Additionally, genetic regulation (genomics), along with other omics platforms, will reveal the biology behind RSA. This review provides detailed insights into the need for RSA studies in crop plants, especially legumes, the genetic mechanisms underlying RSA in relation to multi-omics studies conducted on legume RSA, and future directions for this research area.

## 2 Need to explore nutrigenomics research on legumes for nutritional security

Legumes belong to the family Leguminosae, also called Fabaceae. After cereal crops, Leguminosae are the second most important crops in terms of area and production, providing a low-cost protein source for people worldwide (Farooq et al., 2018). Food legumes make up nearly 27% of global crop production (Ulla et al., 2019). They are a crucial part of the food chain, consumed by both humans and animals, providing plant-based proteins and supporting human health (Kumar et al., 2022). As global food demand is expected to grow (Loboguerrero et al., 2019; Rani et al., 2022; Raimi et al., 2017), it is important to improve and sustain agricultural systems. Legumes are a key source of vitamins, minerals, carbohydrates, dietary fiber, proteins, and phytochemicals (Carbas et al., 2023; Kan et al., 2017; Brummer et al., 2015).

Genomic studies on legume crops have identified a wide array of genomic loci linked with various nutrient traits. Upadhaya et al. (2016) discovered chickpea Quantitative Trait Loci (QTLs) associated with iron and zinc through genome-wide association studies (GWAS). A related investigation was conducted on soybean seeds by Ning and colleagues (2015). The application of next-generation sequencing (NGS), multi-omics, and genomics has significantly contributed to improving the nutrient content of grain legumes and other crops (Banerjee and Roychoudhury 2021; Zargar et al., 2022). Legumes are low glycemic index foods due to resistant starch, non-absorbable carbohydrates, and oligosaccharides (Carbas et al., 2023). Their low glycemic index and slow-digesting starch may help prevent sudden blood sugar spikes and support weight management, making them particularly important for reducing many health conditions (Becerra-Tomás et al., 2018). Additionally, flavonoids, phenolic acids, and tannins in legumes are associated with anti-inflammatory, anti-carcinogenic, antioxidant, and gastrointestinal health benefits (García-Lafuente et al., 2014). Consuming legumes can lower total cholesterol and reduce the risk of cardiovascular disease (Pérez‐Ramírez et al. et al., 2018), as well as decrease triglyceride and low-density lipoprotein (LDL) cholesterol levels by 10% to 15% (Hosseinpour-Niazi et al., 2015). Rich in folic acid, legumes may also help lower the incidence of ischemic heart disease by 16% and stroke by 24% when a higher daily folate intake (0.8 mg) is maintained (Winham et al., 2018). Due to their vital role in disease prevention, legumes are considered “health foods” (Zhong et al., 2018; Kouris-Blazos and Belski, 2016). Therefore, legumes are essential functional foods offering health benefits beyond basic nutrition.

## 3 Understanding abiotic stress responses: impact of drought stress on legumes

Abiotic stresses, including drought, high temperature, salinity, and cold, continuously affect plants and impact the grain yield of legumes and cereals (Zandalinas et al., 2018; Zhou, 2025). Legumes are mostly grown in rainfed production systems and are more vulnerable to drought stress (Zhu et al., 2021). Drought stress affects yield capacity, plant growth, and the content of essential elements, including minerals and nutrients in legumes (Sanusi et al., 2025). Drought can impact crops and hinder growth and grain yield at any stage, but terminal drought is particularly severe (Pushpavalli et al., 2014).

Drought stress impacts photosynthetic machinery, ultimately reducing the yield of legumes, as this process is essential for plant growth and development (Andrianasolo et al., 2016). It affects photosynthesis by altering the fixation of carbon through disrupting various enzymatic activities like PEPCase, Pyruvate, phosphate dikinase (PPDK), Fructose-1,6-bisphosphatase (FBPase), and Ribulose-1,5-bisphosphate carboxylase/oxygenase (Rubisco) (Xu et al., 2025). In soybeans, drought has been linked to a decrease in net photosynthesis, leading to fewer pods and decreased dry matter accumulation, which results in lower overall yield (Sarkar et al., 2021). Irar et al. (2014) identified 18 proteins controlled by the nodule that form a signaling pathway involving the pea and rhizobium genomes, which restrict nitrogen fixation during drought conditions. Qiu et al. (2025) used combined metabolomics and proteomics approaches to explore how metabolites are expressed and regulated in soybeans under drought stress.

To optimize the RSA fitting to specific environmental challenges, the root traits are most often aligned to ideotypes fitting to circumvent the stress conditions. The alignments of root associated traits for strategy to balance the trade-offs between branching in roots, depth of roots and total metabolic cost (Zhang et al., 2026). The nutrient and water acquisition often found in deeper soils grossly depends on alignments of steep, cheap and deep traits. The steep i.e., high root angles help in penetrating deep sol profiles quickly (Afonso et al., 2025). Whereas, cheap roots often possessing low metabolic costs, are often characterized by high cortical aerenchyma that helps to reduce carbon needed for maintenance of RSA (Kaur et al., 2026). In addition, deep root system prioritizes the seminal and primary root elongation over lateral branching in the topsoil. Additionally, plants must align anatomical structure with physiological needs to create balance between resource capture and transport (Kaur et al., 2026). This is accomplished by traits such as thicker pioneer roots that act as highways of root system aligned for structural strength to push roots through compact soil layers. In addition, short lived fine branching roots that act as active miners are efficient for nutrient and water acquisition (Afonso et al., 2025). More importantly RSA traits aligned to biotic and abiotic resilience is accomplished by rhizosphere engineering with microbes aiding in solubilizing minerals and providing essential metabolites (Ren et al., 2026).

To improve the plant’s ability to access higher soil moisture levels, it must develop deep root systems, extensively branched roots, and increased root biomass (Afonso et al., 2025). The benefits of having proliferative and deep-rooting systems, which play a crucial role in drought resistance, are reported across a variety of mainstream crops, such as wheat, maize, chickpea, rice, and soybean (Khan et al., 2025). Additionally, the distribution and diameter of metaxylem vessels that facilitate root conductivity in legumes are also vital for drought tolerance (Purushothaman et al., 2013). Wider root angles help reduce energy expenditure during deep soil penetration in drought conditions (Xiong et al., 2025). Similarly, proliferative rooting systems—including root length density, root surface area and volume, and lateral root number—exhibit enhanced water uptake efficiency in drought-affected soils. For example, in chickpea, the proliferative root system demonstrably improves yield and drought tolerance in water-scarce soils (Basavaraj et al., 2025; Jaganathan et al., 2015). These findings highlight that root system architecture (RSA) is a key factor in overcoming drought stress and represents a significant area for further research to boost crop yield and drought resilience.

## 4 Effect of drought on legume RSA

Roots are the primary plant organs that sense changes in soil moisture. They adapt to these changes at molecular, anatomical, and morphological levels by modifying RSA traits (Amtmann et al., 2022). The response of RSA traits to drought involves a complex regulatory network of sensing, signaling, and gene expression. Ranjan et al. (2022) reviewed that plant hormones play a critical role in the molecular regulation of RSA traits under drought stress. Studies show that under drought conditions, roots adjust their distribution to access water from different soil layers (Song et al., 2020). The distribution of root length density (RLD) across various levels determines how efficiently water and nutrients are absorbed, and research has demonstrated how RLD responds to drought in many crops, including chickpea (Hamedani et al., 2020). These studies indicate that various strategies are employed in response to drought, which often results in a greater detrimental effect on RLD in the topsoil. QTLs linked to key root traits—such as increased root length density, root volume, maximum root depth, and higher root biomass have been identified in grain legumes like chickpea (Kale et al., 2015), common bean (Asfaw and Blair, 2012), and soybean (Manavalan et al., 2015) under drought stress. However, progress in drought-stress reduction efforts for grain legumes has been inconsistent (Deshmukh et al., 2014). One reason for this slow progress might be the complex genetic architecture of drought tolerance, which is mediated by many modest-effect genomic regions and significant interactions between genotype and environment (G×E) (Mosalam et al., 2025). For crop improvement, it is crucial to comprehend the RSA regulatory mechanisms (Wasaya et al., 2018). In response to climate change, developments in OMICs technology over recent years have revolutionized plant breeding and become powerful tools for crop conservation. These OMICs techniques, including next-generation sequencing (NGS), transcriptomics, proteomics, and metabolomics, have been utilized in legumes under abiotic stress.

### 4.1 Why target roots and investigate the RSA?

**Figure 1:**
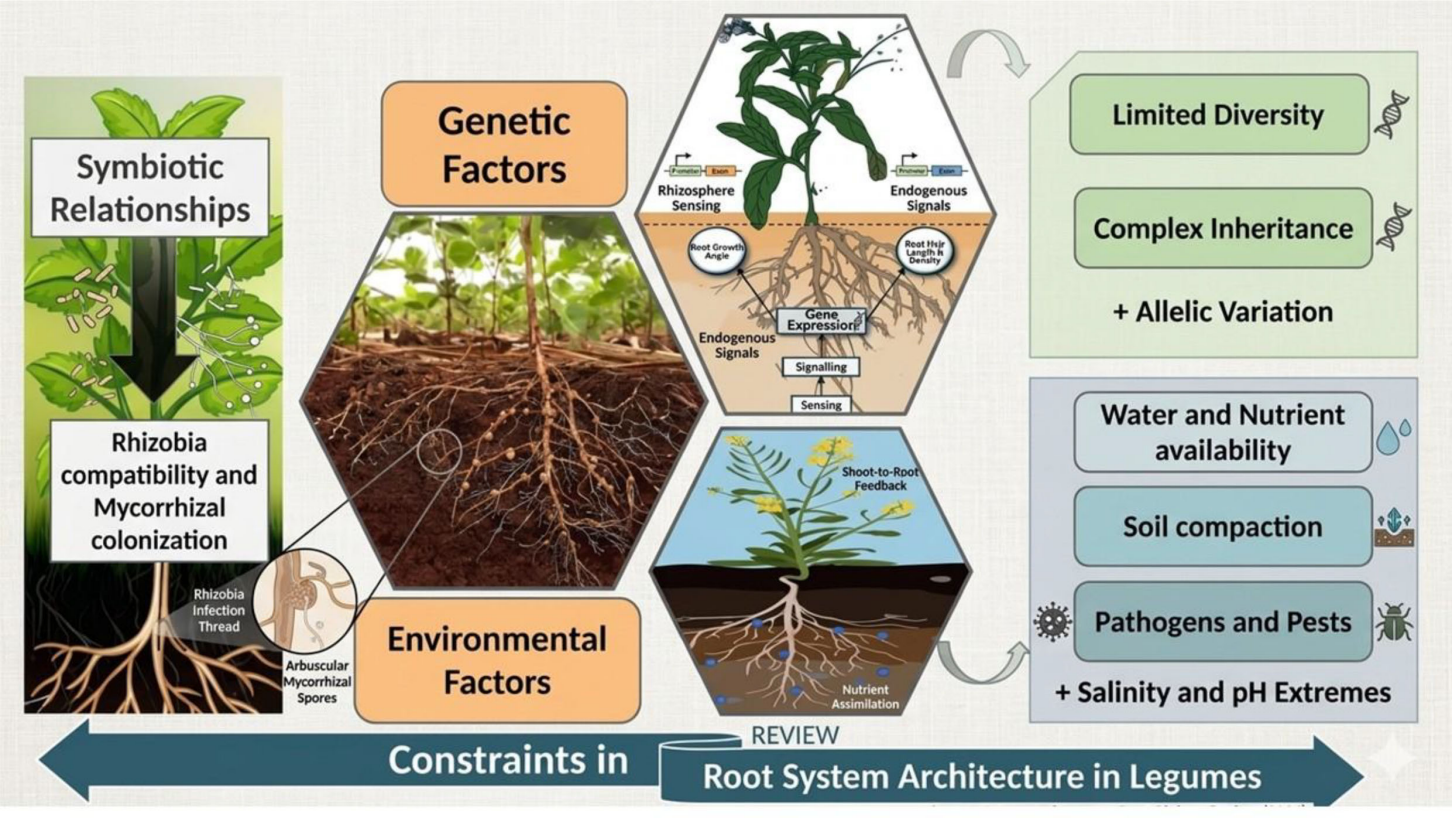
The image illustrates the complex interplay of external and internal factors that may modulate the spatial distribution and root development in legume roots. As depicted the edaphic and environmental constraints such as, physical soil barriers including the fluctuating water table and soil compaction affect lateral root branching and root elongation. Additionally, the chemical stressors such as, deficiency of phosphorus (P) and aluminum (Al) also influence the growth of deep-rooting phenotypes. Figure also demonstrates the significance of high metabolic costs of maintaining symbiotic nitrogen fixation (SNF) apparatus. Genetic and hormonal bottle necks play pivotal role in modulating the expression of genes critical for resistant RSA with higher plasticity. There is critical need for integration of 3D transport mechanism modelled through use of non-invasive imaging techniques to unravel the real time constraints on water flux and nutrient uptake under changing environmental conditions.

RSA are critical for plant growth and development, contributing to various physiological functions such as nutrient and water uptake, anchoring plants in specific soils, storing energy in the form of biomolecules (Abdulraheem et al., 2025). Root systems also help to establish both symbiotic and non-symbiotic relationships with microorganisms (Badhon et al., 2021). Additionally, root systems have made an enormous contribution to ecologically stabilizing the structure of diverse soils, thus preventing soil erosion, retention of water, and facilitating gas exchange (Tron et al., 2015). Secondary functions of root systems include hormone synthesis and storage of photoassimilates. From an ecological perspective, root systems regulate water transport within the soil-plant-atmosphere continuum (SPAC) to approve their role in water recycling. Expanding root systems promotes protecting slopes and promoting good soil consolidation (Bai et al., 2021). Their dynamic and adaptable nature enables plants to rapidly absorb critical soil resources across a wide range of soil conditions, responding effectively to environmental changes (Afonso et al., 2025). Because roots are in direct contact with soil, traits such as root topology, branching angles, link radii, and root length reflect long-term interactions between soil components and root systems (Dhanapal et al., 2021). Consequently, the ability of a plant to explore soil for nutrients and water largely depends on the three-dimensional spatial organization of its root system, known as RSA (Colombi et al., 2018). RSA is also influenced by microbial feedback that is recruited under abiotic stress conditions (Castrillo et al., 2017). Therefore, root morphology and RSA are adaptive responses to soil pressure, heterogeneity, and resource-driven changes in plant morphology and physiology. Several constraints in RSA for legumes are illustrated in **Figure 1**. It is important to note that microbe-mediated RSA restructuring is an intriguing yet underexplored area, requiring further research at the field level using cultivated crops.

### 4.2 Legume root phenomics

Understanding how root dynamics interact with the environment mainly relies on root phenotyping (Bullock et al., 2017). The root dynamics includes the temporal and spatial changes occurring in root system architecture, including its growth and turnover under the influence of changing dynamic environmental conditions (Li Q. et al., 2023). Understanding these dynamics is pivotal to enhance water use efficiency, nutrient uptake and overall resilience of plants to stressors. Unlike the relative fixed developmental cues in many cereals, the legume roots are subjected temporal dynamics through rapid phase transitions and frequent hormonal oscillations to balance the metabolic costs for nutrient and water acquisition (Daryanavard et al., 2026). On the other hand, the spatial dynamics of legumes is exhibited by alteration in growth angle to exploit the resources from soil strata. The root dynamics under stress is governed by rapid signaling networks induced by phytohormones and changing environmental cues (Panozzo et al., 2025). For instance, the scaffolding of abscisic (ABA) and Brassinosteroid (BR) control the speed of root elongation, rate of cell expansion under drought stress conditions (Panozzo et al., 2025). Moreover, proteases and chaperones facilitate the rapid degradation of growth by promoting proteins and enhance the folding under stress conditions thus allowing the RSA modulation fitting to alleviate the heat and salt stressors (Figaj, 2025). Hence exploring the root dynamics associated with RSA is critical and emerging research area to develop crop plants with higher yields and resilience to stressors.

Since RSA influences a plant’s ability to absorb nutrients and water, root phenotyping has gained enormous impetus compared to shoot genotyping under abiotic stress (Afonso et al., 2025). Legumes adjust to different soil conditions for water absorption depending on the thickness of their root xylem vessels (Ramamoorthy et al., 2013). Among various root phenotypes, those exhibiting the most advantageous traits for stress resistance—such as efficient resource acquisition and use—are regarded as superior and higher performing (Afonso et al., 2025). Advances in physiological and genetic research have led to the development of numerous high-throughput technologies for screening these traits (Iyer-Pascuzzi et al., 2011). Phenotyping under controlled conditions, in laboratories or greenhouses, involves both destructive and non-destructive sampling methods to analyze root traits (Angidi et al., 2025).

Various software tools have streamlined root image analysis, enabling quick and reliable phenotyping platforms, including Smart Root, Image J, EZ-Rhizo, WinRhizo, Root Nav (Ratna-Raj et al., 2025), Root System Analyzer (Pierret et al., 2013), Root Trace (Clark et al., 2013), among others. These systems produce root phenotyping data either manually or through imaging, where high-resolution cameras or scanners capture root characteristics like total root length, branching angles, lateral root resolution, individual root diameter, and branching patterns (Angidi et al., 2025). There is no denying that screening for root traits under natural field conditions reveals true trait expression (Iyer-Pascuzzi et al., 2011). However, phenotyping and applying current phenomics technologies for RSA studies in such environments are tedious tasks (Perkons et al., 2014). The most suitable root phenotyping methods identified under field conditions are the soil core method and the standard excavation method, used to study root angle, depth, and density (Perkons et al., 2014). Despite their wide application, these methods have limitations, including crop destruction during root sampling and high labor requirements (Bohn et al., 2006). Therefore, shovelomics is the most commonly used method for collecting root samples in the field (Chen et al., 2014). The mini-rhizotron system has also been used for field studies to monitor root growth, with plexiglass tubes fitted with a scanner or small camera inserted into the soil. Minirhizotrons assess a limited number of genotypes (Riley and van Santen, 2025) and are restricted to small surface areas, but they are mainly employed to study root cortical aerenchyma or water deficiency (Afonso et al., 2025). Another high-throughput technology utilizing non-invasive imaging for RSA studies and root development is Rhizotubes (Jeudy et al., 2016). The total root biomass response to applied current can be measured using the electrical capacitance technique (Manimekala et al., 2025), where two electrodes are inserted at the base of the stem and in the rooting medium (Dietrich et al., 2013). Although this high-throughput phenotyping technique helps in understanding the interactions between soil and roots and in selecting better root phenotypes, it fails to provide detailed information about root architecture, anatomy, and function under field conditions, as observed in soybean (Afonso et al., 2025). X-ray computed tomography has emerged as an excellent non-invasive root phenotyping tool in legumes, especially cowpeas and common beans, enabling detailed 3D RSA analysis in soil cores (Wasaya et al., 2018). This method involves root excavation from soil, washing the roots before imaging, and then analyzing them using multiple commercially available software (Wasaya et al., 2018). Another similar high-throughput, non-invasive technique for studying 3D root system architecture and soil-root interactions is magnetic resonance imaging (MRI) (Metzner et al., 2015). With advancements in non-invasive root trait phenotyping, breeders are gradually shifting their focus from destructive to non-destructive methods for RSA studies. However, the main limitation to adopting such techniques is the significant resources, effort, and expertise required to incorporate them into crop breeding programs, especially in poorer nations.

### 4.3 Root and root system architecture in legumes

Legume roots can develop two different types of secondary structures: lateral roots and nitrogen-fixing nodules. While lateral roots are common in all higher plants, nodules are specific to legume roots, resulting from their symbiotic relationship with nitrogen-fixing soil bacteria called rhizobia (Gonzalez‐Rizzo et al., 2009). RSA in legumes involves the arrangement and structure of roots within the plant, playing a key role in their ecological adaptation and agricultural productivity (Pélissier et al., 2021). The taproot serves as a central anchor for the plant, providing stability and support while also accessing nutrients and water from deeper soil layers. However, RSA in legume plants is not determined solely by the taproot system. It is also affected by the plant’s ability to form lateral roots, which depends on environmental factors such as nutrient availability, stress, or pathogens (Lynch, 2019). Another vital part of legume root architecture is the presence of nitrogen-fixing nodules. These nodules develop during nitrogen deficiency in response to soil symbiotic bacteria collectively known as “rhizobia,” which can fix atmospheric dinitrogen into a form usable by plants. These nodules harbor the bacteria, enabling them to convert atmospheric nitrogen into ammonium that the plant can use for growth (Mohd-Radzman et al., 2013).

Several studies have reported increased activity of a potassium (K^+^) transporter in the mycorrhizal roots of *Lotus japonicus* (Berruti et al., 2016). Mycorrhizal associations increase the effective root surface area for nutrient absorption and improve nutrient mobilization in the soil, thereby enhancing the nutrient acquisition efficiency of legumes (Sui et al., 2022). The updated information on RSA studies in legumes has been highlighted in **Table 1**.

**Table 1:** Updated information on RSA studies in major legume crops.

Legume species	Root trait	Description of study	Significance of root trait studied	Reference
Soybean	Fibrous roots	QTLs identified using recombinant inbred lines (RILs) population.	Trait found to be associated with drought tolerance	Abdel-Haleem et al., 2011
	Root length and root thickness	QTLs identified associated using RILs population.	Traits have influence on the water and nutrient uptake	Prince et al., 2015
	Lateral root number	QTLs identified using Backcross inbred lines (BILs) population	Traits have influenced both root and shoot growth	Manavalan et al., 2015
	Root average diameter, link average diameter, link average length, total root length, Number of root forks, number of root tips	Identified genomic regions underlying these root traits and also candidate gene analysis revealed identification of genes having role in root development	Traits revealed significant variation and are beneficial for their exploitation in future breeding programs	Kim et al., 2023
	Root length, root dry weight	Root traits phenotyped for their response to phosphorus availability	Contrasting root system sizes showed variable response to phosphorus, with deep rooted genotypes showing highest root phosphorus content	Salim et al., 2023
	Root dry weight, nodule dry weight	QTLs identified using association mapping, also putative candidate genes detected	Root trait found to have role in biomass accumulation in soybean plants	Wang et al., 2023
	Root length, root surface area	Expression of *GmEIL4* gene regulate root response to phosphorus stress by increasing root length and surface area	Root traits found to improve phosphorus uptake efficiency	Yang et al., 2023
	Root length, root surface area, root volume	QTLs identified associated with these traits using inter-specific RILs populations. Candidate genes underlying also detected	Root architectural traits improved drought avoidance in soybean	Prince et al., 2020
	Root dry matter, Root length, root volume, surface area average root diameter	Root traits phenotyped under controlled conditions	Root traits found to help cope water stress conditions	Dayoub et al., 2021
	Root length, root surface area	Root traits phenotyped under controlled conditions to determine effect of terminal drought	Root traits found to be associated with better yield under drought conditions	Gao et al., 2020
	Root dry weight, total root length, surface area, root volume, branching number	Genomic regions underlying root traits identified using association mapping	Efficient root traits have ability to enhance resource use efficiency and adaptation to changing climate	Mandozai et al., 2021
Chickpea	Root length density, root surface area, root dry weight ratio, rooting depth	QTLs identified using RILs population	Root traits have been found to be associated with drought tolerance	Jaganathan et al., 2015
	Root length density and root depth	QTLs identified using RILs and also evaluated for root traits response under terminal drought conditions	These root traits have been identified for improving drought avoidance under moisture stress conditions	Gaur et al., 2008
	Root length density	Phenotyping of chickpea genotypes done in PVC cylinders and field conditions; correlated with yield traits	The root trait found to have important role in coping terminal drought	Kashiwagi et al., 2006
	Root length density and root dry weight	Genotypes phenotyped for soil moisture stress and phosphorus stress under field conditions	Root length density reflected more potential for soil moisture and phosphorus acquisition	Afonso et al., 2025
	Root length	Gene (4-coumarate–CoA ligase 1) identified related to trait	Root trait found to be associated with improved phosphorus acquisition	Thudi et al., 2021
	Root length density, root depth, root dry weight	Introgression of QTL hotspots through backcross breeding	Introgression of root traits in elite variety of chickpea improved drought tolerance	Varshney et al., 2013
	Root hair length	Genetic loci associated with root hair length identified using association mapping	Root hair length was found to be associated with phosphorus deficiency	Kohli et al., 2020
	Lateral root count	Identification of *WIP2* gene, underlying major QTL of lateral root	Lateral root contributed positively to higher yield under drought conditions	Dwivedi et al., 2024
	Root length, root biomass and surface area	Phenotyping for traits done using polypropylene pots under controlled drought and irrigated conditions and comparative Transcriptome analysis revealed upregulated expression of genes related to stress-responsive transcription factors, kinases, root nodulation, and oxylipin biosynthesis	The traits maintained crop performance under drought	Bhaskarla et al., 2020
	Root depth, root density, root biomass, root weight	Root traits were phenotyped under drought conditions and related with yield of chickpea	Root traits contributed to higher yield under terminal drought conditions	Pappula-Reddy et al., 2024
Common bean	Basal root angle	QTLs identified using RILs population	Root trait associated with phosphorus uptake and plant growth	Nirmalaruban et al., 2025
	Root dry weight, root length, total root surface, average diameter	QTLs identified for root traits using RILs	Root traits enhance soil foraging provide adaptation to low phosphorus	Afonso et al., 2025
	Tap root diameter, root biomass, tap root length, basal root number, adventitious root number	Root trait phenotyped under controlled conditions for drought and phosphorus stress	Root traits associated with phosphorus stress enhances top soil exploration and deeper roots are favorable for acquiring water and mobile nutrients	Camilo et al., 2021
	Root length, fine root proportion	RILs phenotyped for root traits under controlled condition for drought tolerance	Root traits found to be associated with drought tolerance and contribute to designing improved genotypes adapted to water stress	Polania et al., 2017
	Root depth, root length, root volume, root diameter, root biomass	Genomic regions identified for root traits using RILs	Root traits enabling increased withdrawal of soil moisture from deeper levels under drought stress	Asfaw and Blair, 2012
	Root length, root dry weight, root diameter, number of root tips	Genomic regions underlying root traits identified using RILs	Better performance of root traits providing the ability to overcome aluminum toxic conditions	López-Marín et al., 2009
	Root length, root area, root biomass, taproot mass	Inheritance of root traits studies and additive genotypic effects detected	Selection for better root traits indicated improved phosphorus uptake	Araújo et al., 2005
	Root angle, root length, root number	Combining ability and heritability of root traits studied using North Carolina II mating design	Better root traits with positive GCS effects have potential to be used for genetic improvement of crop suitable for marginal environments	Castiano et al., 2023
	Adventitious root number, basal root number, primary rot branching	Root traits phenotyped at seedling stage and in field for assessing diversity in core collection	Root traits associated with drought and low phosphorus adaptation	Jochua et al., 2020
	Basal root number, tap root length, adventitious root number, basal root whorl number	Root traits phenotypes at seedling stages and compared with root phenotypes in field across environments	Root traits found to have an influence on adaptation to edaphic stresses	Strock et al., 2019
Cowpea	Basal root angle, root diameter, median width, adventitious root number, average root density, root tissue angle	Diverse panel of cowpea used for QTL identification through association mapping.	Better root phenotype improves the acquisition of soil resources and co-localization QTL for root systems with those of Striga resistance indicating the role of root architecture in avoiding Striga attachment and growth	Rascher et al., 2011
	Root biomass	Effect of rhizobacillus on root under stress conditions detected	Metabolics in root trait triggered by rhizobacillus lead to efficient nutrient allocation	Abiala et al., 2023
	Root dry weight	Genotypes grown under controlled drought conditions and evaluated for root and agro-morphological traits	Plant invests more for root development under drought conditions that aids in providing drought tolerance	Santos et al., 2020
	Root dry weight, hypocotyls root length, lateral root number, root branching density, root diameter	Root traits phenotyped under field conditions and measure genotypic variation and identify candidate variables	Root traits contributing to variations provide considerable sources for improved cowpea genotypes favorable for marginal environments	Adu et al., 2019
	Root dry matter, root length, tap root length	Root traits phenotyped under controlled conditions for understanding effect of drought	Root traits have role in providing drought tolerance	Matsui and Singh, 2003
	Adventitious root number, basal root angle, basal root hair density, root length, tap root branching, tap root hair length	Root traits phenotyped at seedling stage under controlled conditions for evaluating root architecture variations	Better root growth improve the phosphorus acquisition under low phosphorus conditions	Mohammed et al., 2022
	Root length, root volume, root biomass, root density, root dry matter	Root traits evaluated and associated with yield traits to explore improvement potential of crop	Root traits not much significantly related to yield in cowpea as its root does not require robustness to absorb water and nutrients	Ittah and Arua, 2017
	Root length, root volume, dry root weight, root biomass density, tap root diameter, adventitious root number	Identification of genomic regions underlying the root traits of diverse cowpea mini-core collection using genome wide association studies	Genotypes with promising root traits improve acquisition to soil resources particularly under marginal environment	Zaffar et al., 2024
Mungbean	Root length, root surface area, root volume, root average diameter, total root tips, root forks	Root traits phenotyped under optimum and low phosphorus conditions to observe variations	Root traits significantly varied for response to phosphorus uptake efficiency at seedling stage	Reddy et al., 2020
	Root dry weight, root length, root surface area, root angle, root collar diameter, number of root tips	Root system architecture phenotyped to explore its association with above- ground growth and development	Desirable root traits could be combined to target different production environments. Deeper, longer roots for exploiting deep reserves of water and nutrients and shallow root systems for crops grown in shallow soils	Singh and Bell, 2021
	Root length, root surface area, root diameter, root volume, total root tips, total root forks, root dry weight, total root crossings.	Root trait phenotyped for identifying diversity in mungbean mini-core collection from AVRDC	Better root growth improves the uptake of water to mitigate drought and heat stress	Aski et al., 2021
	Root length, lateral root branches, root surface area, root diameter, root volume	Root traits phenotyped for diversity analysis and association studies done to identify genomic regions underlying	Better root traits provide drought tolerance and root-based ideotype categories useful for the improvement of crop for stress conditions	Chiteri et al., 2022
	Root mass density, root distribution percentage	Root traits phenotyped under controlled conditions to examine effect of phosphorus and potassium application	Root traits maximize fertilizer use efficiency when applied in rhizosphere zone	Htwe et al., 2023

The plasticity is central weapon for plant survival and modulatory key under changing environmental conditions. The plasticity of RSA greatly enhances the plants’ ability to dynamically alter the architecture in response to environmental cues (Kaur et al., 2026). Reports suggest that legumes possess a high degree of phenotypic plasticity, even a single genotype can produce diverse root structures depending on the severity of drought stress (Afonso et al., 2025). For instance, the legumes trigger deep mining strategy by suppressing the growth of lateral roots in dried topsoils to conserve the carbon under drought stress (Ye et al., 2018). Additionally, by slowing the leaf expansion and accelerating root exploration, the plant prioritizes the water acquisition over vegetative bulk (Kaur et al., 2025). The metabolic plasticity though cortical aerenchyma reduces the cost of maintaining the RSA, thus allowing plants to sustain the larger root systems with utilization of less energy (Kaur et al., 2025). Thus, plasticity allows legumes to be “smart” foragers rather than just maintaining a rigid structure under dynamic environmental conditions.

### 4.4 Omics approaches for analyzing legume roots in RSA studies

Omics techniques are widely used to explore the regulatory landscape that controls the growth and development of legume roots under normal and extreme environmental conditions. Specifically, root genomics involves studying the entire set of genes expressed (the genome) in legume roots. This mainly includes identifying genes involved in root development, architecture, nutrient uptake, stress responses, and symbiotic interactions with rhizosphere organisms. Genomic methods, such as high-throughput sequencing, genome-wide association studies (GWAS), Recombinant Inbred Lines (RILs), QTL mapping, and functional genomics (studying gene functions), are employed to uncover the genetic basis of RSA in legumes (Salgotra and Stewart, 2022). Therefore, discovering new QTLs is crucial, as it provides a key research pathway for exploring the extensive genetic variability related to root system traits. The development of GWAS has greatly improved the ability to identify genes that control RSA-related traits. Using genome-wide mapping techniques, researchers have identified new genetic loci associated with root architectural traits in legumes, utilizing various mapping populations, including introgression lines, RILs, biparental populations, and global core collections (Siddiqui et al., 2021).

In a study, high-density single-feature polymorphic markers and simple sequence repeats (SSRs) were used to map QTLs controlling RSA in an interspecific soybean mapping population derived from a cross between *Glycine max* and *Glycine soja* (Prince et al., 2015). QTLs for three different traits, such as CaLG04 for root length density, CaLG06 for root surface area, root dry weight, and CaLG04 for total plant dry weight, were identified in chickpea for drought tolerance (Fan et al., 2021). Similarly, in common beans, sixteen QTLs have been mapped for root gravitropic traits, including three for basal root angles (Brg1.1, Brg5.1, Brg5.2), which are related to deep rooting (Joshi et al., 2022) through QTL mapping. Idrissi et al. (2015) studied the variation in root traits associated with drought tolerance using an F6–8 population of 133 RILs derived from the cross ILL6002 × ILL5888. QTL mapping was performed in a population of pea RILs resulting from a cross between P665 and Messire of Pea. Three QTLs linked to root length were identified on chromosomes (Chr.) 3 and 4, where they overlapped with QTLs connected to weed and fungal resistance (Ye et al., 2018). The improvement of various root traits in legume crops, including chickpea varieties JG 11 and JG 130, has been achieved through Marker-Assisted Backcrossing (MABC)/Marker-Assisted Backbreeding (MABB) approaches (Salgotra and Stewart, 2022). GWAS has been performed in different studies to identify the underlying genes and QTLs related to root structure in legumes (Susmitha et al., 2023). Soybean accessions with minor and mutated allelic variants of the lateral root number (LRN) gene and the distribution of root diameter in Class I, along with a major locus on chromosome 16, were found to perform better under both water-limited and optimal field conditions (Susmitha et al., 2023).

A significant association of SNPs was observed between common bean root traits and the toxic compound extrusion gene, as well as aluminum. This association activated the malate transporter gene, contributing to tolerance to aluminum toxicity (Ambachew and Blair, 2021). Reverse genetics have also been used to identify several genes that may play a role in altering root architecture in response to phosphorus (Pi) deficiency (Yang et al., 2021). For instance, in soybeans, GmEXPB2 is inherently linked to root system architecture responses to various abiotic stresses. Overexpressing GmEXPB2 led to modifications in soybean root architecture by increasing root hair density and enlarging the root hair zone (Chen et al., 2011). Genomic studies are crucial in investigating traits related to RSAs in legume crops.

In addition to molecular analysis several reference genome versions are employed to identify the candidate genes governing RSA in crop plants (**Table 2**) (Gaiwal et al., 2026). For instance, 14 candidate genes were identified to influence the RSA in pigeon pea. These include gene *C. cajan_07827* which encodes for the *unc-50 like protein*, gene *C. cajan*_00496 codes for a *LOB domain-containing 22 protein*, *AT-hook motif nuclear-localized protein 15–29 protein encoding gene C. cajan_13135* and gene *C. cajan_21512* which encodes for a *root cap* protein family. Similarly, in *Medicago truncatula* several genes have been identified to modulate the RSA linked to primary root elongation and lateral branching. For instance, CRA1 (Compact Root Architecture 1) regulates lignin biosynthesis and aids in polar transport of auxin thus directly regulating the cell wall metabolism to modulate RSA. The MtHB1 (Homeobox 1) encoding HD-Zip1 transcription factors expressed by salinity stress helps to extend the lateral root emergence for prioritizing the deep taproot growth over surface branching during salinity and drought stress. The genes like NSP1 and NSP2 coding for GRAS transcription factors show they interact with SHR (shoot root) and SCR (Scarecrow) pathways to modulate the radial patterning of roots important for cortical cell division and symbiosis.

**Table 2:** This table summarizes key candidate genes critical for modulating RSA in legumes.

Gene ID	Protein product	Biological process regulated	Molecular function	RSA trait	References
*C.cajan_00496*	LOB domain-containing 22	Increases drought tolerance by regulating key stress-related pathways, including antioxidant activity and root development	DNA-Binding Transcription Factor	Tap root length	Gaiwal et al., 2026
MtG\beta 1	G-protein Beta Subunit 1	It helps scaffolding and signal transduction and interacts with G-alpha and G-gamma subunits.	Helps in modulation of abscisic acid (ABA) and brassinosteroid signaling pathways.	Primary Root Length & Vigor:	Sun et al., 2025
*C.cajan_13135*	AT-hook motif nuclear-localized protein 15–29	Helps in vegetative to reproductive phase transition of meristems	It is a DNA-binding transcription factor, minor groove of adenine–thymine-rich DNA binding	Tap root length	Gaiwal et al., 2026
PIN Family	Auxin Efflux Carriers	It is a transmembrane transport of auxin; regulates direction and rate of cellular auxin exit	Helps in establishing auxin gradients and “auxin maxima” in root tips and primordia.	Root Gravitropism & Branching	Jha et al., 2025
*C.cajan_07827*	unc-50 like	Aids in transport mechanism	-	Tap root length	Gaiwal et al., 2026
*C.cajan_02617*	SNARE-interacting KEULE-like isoform X2 protein	Helps in vesicle docking involved in exocytosis	-	Number of lateral roots	Gaiwal et al., 2026
*C.cajan_22424*	Glyma.20G191900, Vigun07g189600, Phvul.007G140800, AT5G04420	acyl-binding domain-containing 4-like protein	–	Root diameter	Gaiwal et al., 2026
*MtCRE1*	Cytokinin Receptor 1 (Histidine Kinase)	Possess high-affinity binding of cytokinins and triggers a phosphorylation signaling cascade.	It regulates cortical cell division and suppression of lateral root initiation.	Lateral Root Density:	Ariel et al., 2012
*C.cajan_06237*	Protein fluG	It aids in glutamine biosynthetic process	It is a glutamate-ammonia ligase activity, hydrolase activity	Lateral root length	Gaiwal et al., 2026
*C.cajan_20841*	BAG family molecular chaperone regulator 6	-	Helps in folding since this protein has chaperone binding	Lateral root length	Gaiwal et al., 2026
*C.cajan_22050*	aspartic protease	Helps in proteolysis	Has aspartic-type endopeptidase and transferase activity	Lateral root length	Gaiwal et al., 2026
*C.cajan_11792*	Protein MAINTENANCE OF MERISTEMS-like (IPR044824)	Regulates meristem maintenance and meristem development	-	Lateral root length	Gaiwal et al., 2026

### 4.5 Proteomics for investigating RSA in legumes

Proteomics is another powerful omics tool for understanding protein expression and interaction in legume roots (Joshi et al., 2022). Techniques like mass spectrometry and protein profiling enable the identification and quantification of proteins involved in metabolic pathways, signaling, stress responses, and symbiosis (Nouri and Komatsu, 2014). The proteomics approach has been applied across various legume species such as soybean, chickpea, cowpea, pigeon pea, groundnut, and common bean, as well as in legume model systems like *Medicago*, for identification of proteins associated with specific RSA traits (Gifford et al., 2024). Ultimately, these insights hold significant promise for advancing sustainable agriculture practices and improving crop yields.

For instance, protein quantification revealed nodule-specific Cu+-chaperone NCC1, which is essential for copper-dependent physiological processes and copper homeostasis in symbiotic nitrogen fixation in *Medicago truncatula* root nodules (Navarro‐Gómez et al., 2024). Chen et al. (2023) revealed differential protein profiles of root nodules of soybean responding to phosphate starvation through proteomic analysis. Upregulation of root cell wall proteins (CWPs), purple acid phosphatase 1-like (GmPAP1-like) in Soybean, which regulate complex changes in the root system in response to P deficiency, were identified by using iTRAQ (isobaric Tag for Relative and Absolute Quantitation) proteomic analysis (Wu et al., 2018; Thal et al., 2018). Defensive proteins, including endoglucanase Protein, Zinc finger CCH domain-containing protein, serine carboxypeptidase, and beta−glucosidase, were identified by Matrix-Assisted Laser Desorption/Ionization Mass Spectrometry (MALDI-MS) and unravel the mechanism that leads to a change in root architecture to adapt Fe and P deficiencies in common bean roots (Thal et al., 2018; Manzoor et al., 2021). Various other proteomics approaches have been employed to investigate changes in legumes’ RSA traits to adapt to various types of stresses (Jan et al., 2023). Thus, proteomic approaches serve as powerful tools for investigating the architecture of legume roots. Moreover, proteomic analyzes offer insights into the regulatory pathways and protein networks that govern RSA in legumes, thereby facilitating the development of strategies to enhance crop productivity and sustainability. Overall, proteomics offers a comprehensive and detailed understanding of legume root architecture, contributing to advancements in plant biology and agriculture.

### 4.6 Metabolomics for investigating RSA in legumes

Metabolites in legume roots influence growth and architecture (Joshi et al., 2022). Under stress conditions, roots adjust metabolomic pathways through acclimation, activating signal pathways, and synthesizing proteins and metabolites to achieve a new equilibrium (Makhumbila et al., 2022). Metabolomics allows precise characterization of root metabolites, revealing changes in response to environmental conditions and stress, impacting RSA in legumes (Joshi et al., 2022). Metabolomics is advancing rapidly, with current emphasis on metabolic fingerprinting and metabolite profiling techniques. To encompass the diverse array of metabolites, various analytical methods employing separation and detection are used in plants (Doerfler et al., 2014). Separation techniques are tailored to specific metabolite groups, gas chromatography (GC) for volatile and primary metabolites like sugars and amino acids, liquid chromatography (LC) primarily for secondary metabolites, capillary electrophoresis (CE) for ionic metabolites, and ultra-performance liquid chromatography (UPLC), which boasts high resolution, sensitivity, and throughput compared to conventional HPLC (Aarika et al., 2025). Mass spectrometry (MS) analyzers are widely utilized for metabolite profiling, particularly those offering accurate mass measurements such as FTICR-MS, Orbitrap-MS, or TOF-MS, due to their swift scan times, improved deconvolution, run times, and high mass accuracy. GC-MS is extensively employed in plant metabolomics research, benefiting from electron impact (EI) for robust interfacing with MS, ensuring highly reproducible fragmentation patterns (Ramalingam et al., 2015).

Metabolite profiling involves measuring all or a subset of metabolites in each sample simultaneously. While the use of metabolic profiling has been limited in crop legumes, this approach has shown success in model legumes. For instance, an untargeted quantitative MS approach was utilized to profile metabolites treated with rhizobial Nod factors to investigate metabolic changes between symbiont roots in *Medicago* (Zhang et al., 2013). Metabolomics played a pivotal role in elucidating the metabolic intricacies of *Medicago truncatula* border cells through mass spectrometry analysis. Significant metabolic differences between border cells and root tips were revealed, with metabolomics identifying key metabolic pathways such as the oxylipin-pathway, lipoxygenases, and auxin-responsive pathways. The presence of starch deposits serving as critical energy reserves in border cells was highlighted (Watson et al., 2015). Moreover, comparative metabolomics between Lotus species revealed the presence of conserved and unique metabolites in response to drought stress and resulted in changes in root morphology (Ramalingam et al., 2015). In another study, alanine accumulation under anoxic conditions was examined in *L. japonicus*, which exhibits high tolerance to waterlogging. High accumulation of succinate, alanine, glutamate, and gamma-aminobutyric acid (GABA), the direct co-substrates for alanine synthesis, was observed in the roots of *L. japonicus* during waterlogging (Rocha et al., 2010). Komatsu et al. (2011) used CE-MS to identify 81 metabolites related to mitochondria under flooding stress in roots and hypocotyls of soybean. Furthermore, studies have explored the role of phosphorus in stress response metabolite profiling of common bean roots and nodules under P starvation (Hernández et al., 2007). They reported an increase in the levels of most amino acids and several sugars in P-stressed roots, suggesting a preferential partitioning of sugars to support the expression of P stress-induced genes (Urwat et al., 2021). Therefore, metabolomics has revolutionized our understanding of root metabolism in legumes. These findings highlight the dynamic relationship between root metabolism and environmental challenges, offering valuable insights into root physiology and its implications for plant adaptation and resilience.

The legume specific metabolites have been identified by using UPLC-Q-TOF MS, a high-resolution untargeted profiling of isoflavonoids, saponins, and signaling peptides (Du et al., 2025; Afonso et al., 2025). Similarly, the derivatized GC-MS has led to identify the primary metabolites such as, sugars, amino acids and organic acids like citrate and malate which are critical for energy flux. Additionally, the real time changes in metabolic concentrations within live roots have been investigated by NMR spectroscopy, a non-destructive metabolomic technique. The heat maps of metabolites pivotal for RSA specific to root cap and infection thread have been investigated by employing MALDI-ToF MSI technique.

Metabolomic techniques led to the identification of wide range of RSA specific primary and secondary metabolites by employing advanced metabolomic techniques. For instance, the flavonoids such as apigenin, luteolin, and legume specific isoflavonoids such as genistein and daidzein identified by MALDI-MSI are very central to regulate the RSA in legumes (Zhang et al., 2025). They play critical role as chemoattractants for *Rhizobium* bacteria and trigger the transcription of *nod* genes important for establishing symbiosis. Moreover, the flavonoids inhibit polar auxin transport and directly influence the initiation and emergence of lateral roots and production of nodule primordium (Zhang et al., 2025). Similarly, the strigolactones in legumes identified through metabolomic approaches play pivotal role in shaping root and shoot architecture (Afonso et al., 2025). They are found exuded into soil to signal the Arbuscular Mycorrhizal Fungi (AMF) to promote the hyphal branching and establishment of symbiosis. The strigolactones suppress the lateral root formation and aid in promoting the root elongation to making plant roots to explore deeper soil layers for transport of water nutrients.

By employing UPLC-QTOF-MS (Ultra-Performance Liquid Chromatography Quadrupole Time-of-Flight Mass Spectrometry) tritepinoids saponins critical for anti-herbivory and antimicrobial activity have been identified in legumes such as, *Medicago truncatula* and *Glycine max*. these metabolites apply critical role in RSA modulations by influencing the root cell division and elongation (Ngoc et al., 2025). They also alter the soil microbial communities and trigger root associated microbes to release volatile organic compounds that trigger the root branning in legumes. Legumes also secrete non-protein aminoacids such as, Canavanine and Mimosine identified by HILIC-MS (Hydrophilic Interaction Liquid Chromatography) in root zone, which act as allelochemicals to reduce competition from non-legume plant species (Seregin and Kozhevnikova, 2024; Afonso et al., 2025). They modulate RSA by inhibiting the root growth pf competitors and ensure the legume RSA expands at its maximum.

### 4.7 Rootomics: an integrated omics approach for RSA studies under optimum and drought conditions

**Figure 2:**
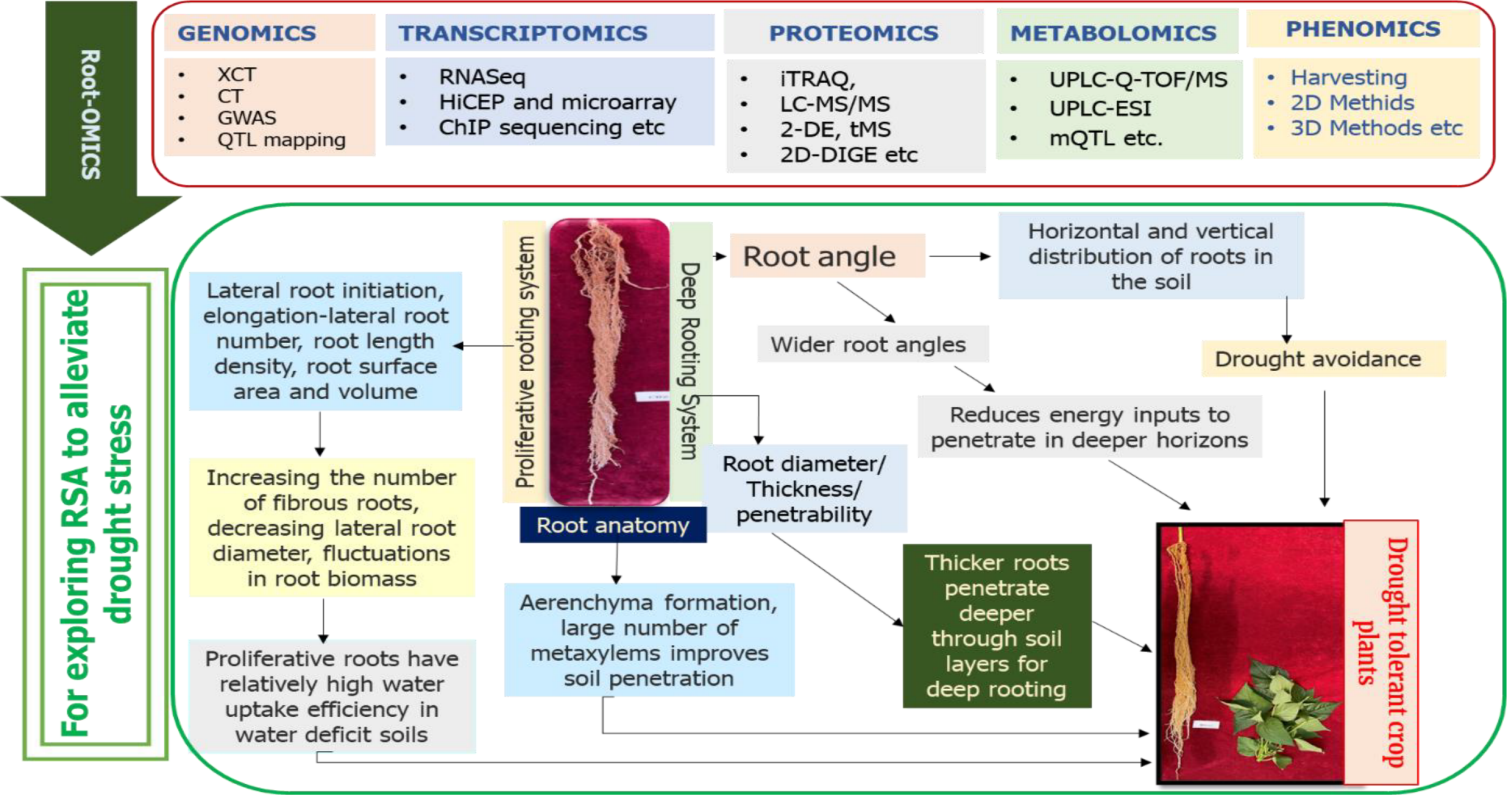
Schematics displaying robotics approaches to unravel the RSAs resilient against drought stress. The RSA attributes like deep rooting are a compound trait largely affected by root angle and root length. The figure further indicates that root angle determines the actual direction of vertical and horizontal root distribution in soils to circumvent drought stress. Further, wider roots are critical for penetrating the deeper horizons of soil to access the deeper waters. Additionally, deep root systems are also determined by the diameter of the root and penetrability. While thicker roots penetrate deeply into the layers of soil to uptake water. The proliferative rooting is another important RSA attribute defined by lateral root initiation and elongation and can enhance the efficiency of crop plants to uptake water in drought conditions. Plants can also modulate their RSA by inducing plasticity to increase the number of fibrous roots, decline lateral diameter, and change the biomass of roots. Finally, the figure also highlights the adjustments of root anatomy in terms of aerenchyma formation to penetrate the soil with the use of much energy and enhance the number of metaxylems to efficiently transport water to aerial parts of plants under drought-stress conditions.

Inheritance studies suggest that numerous traits regulated by a network of genes, including interactions and epistasis, can be modulated in accordance with environmental conditions (Slovak et al., 2014). A wide range of rootomics approaches are currently used to analyze RSA traits under both natural and controlled experimental conditions, including drought stress (**Figure 2**). Presently, rootomics methods such as high-throughput sequencing, GWAS, QTL mapping, and RILs are utilized to examine crop RSA under stressful conditions (**Table 3**). To dissect complex traits, numerous QTLs have been identified that influence RSA traits such as root biomass, root length, and root number in legumes (Kashiwagi et al., 2006). Moreover, various specific genes related to RSA regulation have been reported in crop plants through QTL analysis or genetic mutants with quantifiable traits. For instance, in rice, six QTLs associated with deep-rooting systems were identified under drought-stress conditions (Kitomi et al., 2018). Similarly, in double haploid wheat populations, 23 QTLs that regulate root growth rate under water stress were identified, with seven of these responsible for RSA traits like root number, root length, seminal root angle, and total root surface area (Ma et al., 2017). In chickpea, three QTLs, one each for root surface area (CaLG06), root dry weight/total plant dry weight ratio (CaLG04), and root length density (CaLG04), were identified as controlling RSA (Jaganathan et al., 2015). This research also led to the discovery of a ‘QTL hotspot’ that influences multiple RSA traits and drought stress tolerance (Jaganathan et al., 2015). Still, many QTLs remain to be identified to fully understand the complex gene network responsible for regulating the spatial organization of RSA.

**Table 3:** Root-omics technological advances to explore the RSA in plants.

OMICS approach	RSA type	Technique name	Root studies carried	References
Phenomics	Harvesting method	Digging tool Weighing tool Washing tool	To explore the characteristics, functions, and ecological adaptation strategies of root systems	Augusto et al., 2015
	2D-morphological observation	Pinboards	Root Length, root weight, root diameter and distribution pattern	Judd et al., 2015
		Rhizotrons	Provides dynamic 2D information on growth and turnover, root morphology of primary root, lateral roots, and adventitious roots. Aids in monitoring fine root growth, development, and rate of decomposition	Judd et al., 2015
		Minirhizotrons	Provides dynamic 2D information on growth and turnover, root morphology of primary root, lateral roots, and adventitious roots. Aids in monitoring fine root growth, development, and rate of decomposition	Kilpeläinen et al., 2019
		Mesorhizotrons	Provides dynamic 2D information on growth and turnover, root morphology of primary root, lateral roots, and adventitious roots	Kilpeläinen et al., 2019
		WinRhizo imaging	To assess root density, root angles, appearance, root branching and distribution pattern, root length and root surface area	Clark et al., 2013
		RootTyp model	To assess different root structures and root development stimulation.	Saint Cast et al., 2019
		Horhizotron™	To assess root density, root appearance, root branching, and distribution pattern	Judd et al., 2015
		Shovelomics	To quantify mature field-grown roots To acquire measurements by excavation 2D imaging and automated image processing	York et al., 2018
	3D- image reconstruction	Ground penetrating radar (GPR)	Construction of 3D maps of RSA To study coarse roots (diameter >0.5 cm)	Butnor et al., 2016
		Magnetic resonance imaging (MRI)	To reconstruct RSA in three or four (space and time) dimensions To evaluate the structural-function relationships of root systems	Perelman et al., 2020
		X-ray computed tomography (XCT)	Imparts no damage to the soil, plants, or environment It can characterize the RSA of individual plants or sample plot	Cercioglu et al., 2018
		Neutron computed tomography (neutron CT)	Imparts no damage to plants Helps to characterize the RSA of individual plants or sample plot	Koch et al., 2019
		Genome-wide association studies (GWAS)	To analyze inherited traits exhibiting extensive heritability (For example identification of CLV2 to initiate root meristem differentiation) AtPHR1 gene to control root growth AtVDAC3 to regulate Primary root growth control OsDRO1 to regulateDeep rooting under drought stress	Bouain et al., 2019
		Quantitative trait loci (QTL) mapping	Identification of QTLs such as, qTLRN-12 and qLLRN-12f for lateral root development plasticity and aerenchyma formation improved rice adaptation under increased soil moisture stress. Identification of qDTY3.2 QTL for imparting drought tolerance in rice	Dwivedi et al., 2021
Transcriptomics		RNASeq HiCEP and microarray techniques	Identification of genes for diverse RSA attributes such as, SlPIP1;7 that promotes root growth along with tolerance to drought CaTIP2/3 helps in drought tolerance CaNIP6;3 alleviates Drought tolerance TaPSTOL1 enhances early root growth Ci1-FFT enhances root growth under freezing conditions	Azeem et al., 2019
		ChIP sequencing	Identification of transcription factors critical for root development	Nanjo et al., 2011
Proteomics	Proteome profile	iTRAQ, LC-MS/MS	To analyze signal transduction pathways, Heat shock proteins, secondary metabolites, and lignin metabolism-associated proteins to alleviate drought stress by improving root growth	Alvarez et al., 2014
		2-DE, tandem MS	Analyzed higher lipid peroxidation by malondialdehyde (MDA) at roots. Upregulation of glutathione S-transferase (GST) and downregulation of MDA led to improved Cu stress tolerance via roots	Li et al., 2013
		2D-DIGE	Primary rooting depth was reduced due to the accumulation of oxygen in the root tip and the size of the meristem Inhibition of peroxidases (PODs) activity, brassinosteroid (BR) by TaTRIP1. 24-epibrassinolide increased root meristem size.	He et al., 2014
		2-DE gel, Nano-LC/MS	Assessing root elongation due to upregulation and downregulation of proteins.	Oh et al., 2014
		Q-TOF/LC-MS	Assessing root tip growth under salinity stress by differential amounts of proteins linked to TCA cycle	Dissanayake et al., 2022
Metabolomics	Metabolite profile	UPLC-Q-TOF/MS	About 3127 differentially accumulated metabolites (DAMs) were detected, including amino acids, lipids, and their derivatives, alkaloids, carbohydrates, organic acids, terpenes, coenzymes, nucleotides and derivatives, flavonoids, lignin, alcohols, coumarins, aldehydes, phenolics and ethers	Li C. et al., 2023
		UPLC-ESI-Q-TOF-MS	To analyze the regulation of membrane lipid turnover, release of secondary metabolites to prevent lipid peroxidation by scavenging ROS and chelation of metal ions	Zhu et al., 2021

Through GWAS analysis, candidate genes responsible for variations in root growth rate under stress conditions are identified. For example, GWAS has been used to locate gene loci that influence root growth rate, which is essential for alleviating deficiencies of P, Fe, and Zn in Arabidopsis (Kang et al., 2008). This approach identified the CLV2 gene product as a promoter of CLE14 expression, initiating root meristem differentiation under P deficiency (Gutiérrez-Alanís et al., 2017). The genes controlling root system architecture (RSA) has also been studied using transcriptomics. For instance, in rice, several genes encoding transcription factors and other metabolites are responsible for regulating RSA (Song et al., 2016). Transcriptomic studies have identified candidate genes that control RSA under drought conditions in grain legumes (Bhaskarla et al., 2020). Similarly, in chickpeas, RNA-seq analysis has uncovered genes that contribute to drought tolerance (Mahdavi Mashaki et al., 2013). Micromics, another rootomics technique, is used to explore the collection of short 20–24 miRNAs involved in post-transcriptional regulation of RSA-related genes in plants (Ding et al., 2013). These small non-coding RNAs are key regulators of RSA under drought stress in both leguminous and non-leguminous plants (Couzigou and Combier, 2016). For example, miRNAs such as miR393, miR160, miR167, miR164, and miR171 regulate nutrient availability through RSA modulation (Zhakypbek et al., 2025). Additionally, miR828 targets the S15 MYB transcription factor that controls root hair patterning in apples (Xia et al., 2012). Numerous studies suggest that miRNAs are crucial in RSA regulation by targeting genes involved in responses to rhizosphere complexity and nutrient availability. However, much remains to be explored regarding the diverse roles of miRNAs in modulating RSA under drought stress.

Proteomics is another forefront rootomics approach employed for the identification and interaction of proteins temporally important for studying RSA. This approach helps to identify modifications operated at stages of translation and post-translation, thus enhancing our understanding of RSA under stress conditions (**Table 3**). Gupta et al. (2020) used two-dimensional gel electrophoresis (2-DE) and Matrix-Assisted Laser Desorption/Ionization Time-of-Flight (MALDI-TOF MS/MS) proteomic techniques to unleash the complexity of proteomes in roots under drought stress conditions.

Metabolomic studies help unravel the intermediate and end products of metabolic pathways pivotal in regulating the expression of phenotypic traits critical for RSA under drought stress conditions (Khan et al., 2019). These studies strongly support a large number of metabolic pathways such as those concerned with sugar metabolism and the ethyl/auxin pathway produce metabolites controlling root traits under drought stress conditions. For instance, comparative root metabolomic studies of non-legume crops and legume crops carried out under drought stress identified root-specific accumulation of metabolites such as 4-hydroxy-2-oxoglutaric acid (tobacco roots) and coumestrol (soybean) (Rabara et al., 2017). These metabolites are reported to control the RSA in both categories of these experimental plants. Very few studies have been conducted on the role of metabolites in regulating RSA.

**Figure 3:**
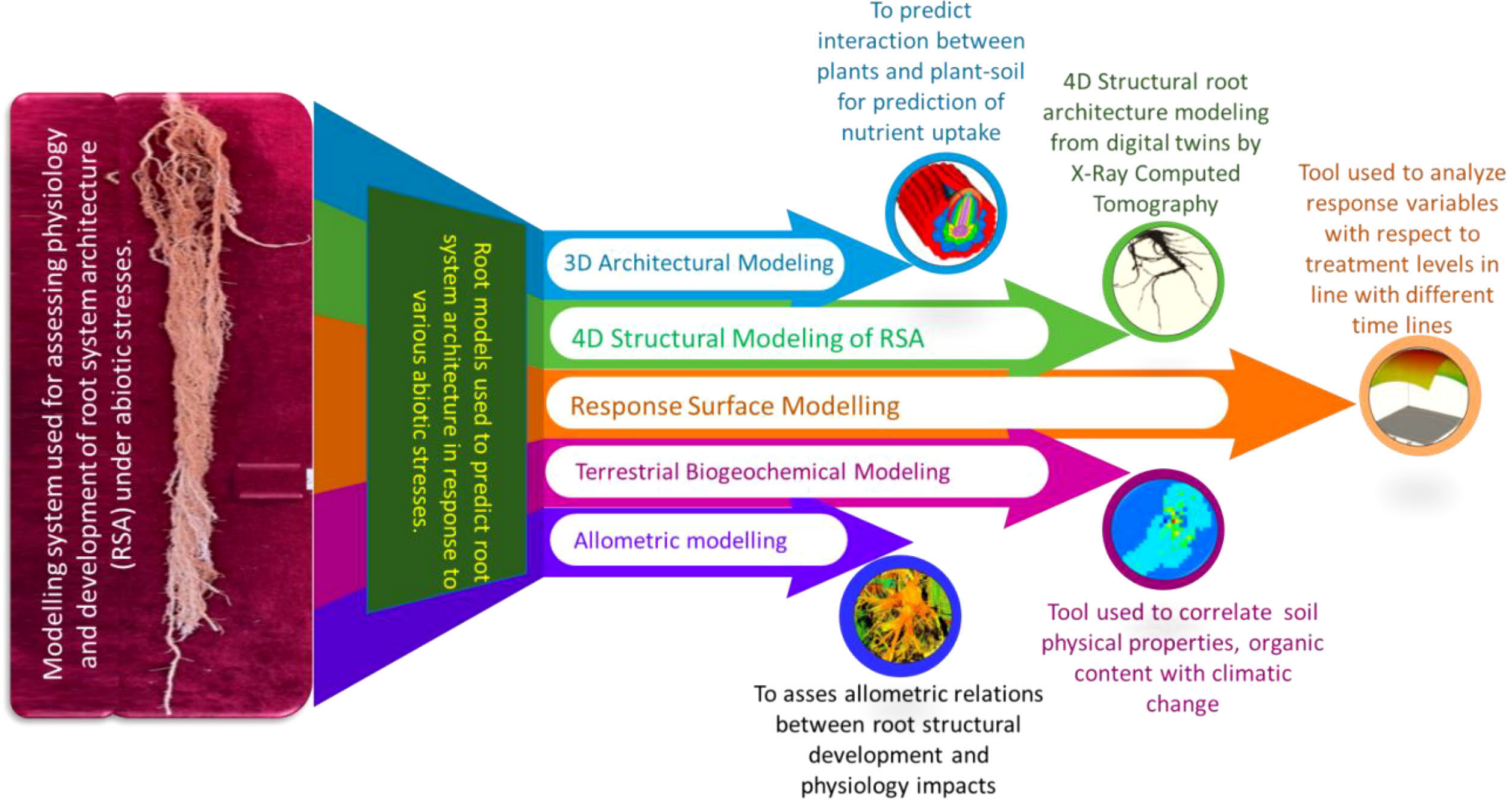
Demonstration of various root modeling tools employed to predict root system architecture in response to exposure to abiotic stresses.

In recent times, rootomics analysis based on phenomics has been a common method to understand the phenotypic traits of root growth and development for RSA analysis. Over the past decade, several high-throughput techniques have been developed for conducting phenomic studies to explore RSA in legumes. High-throughput phenotyping (HTP), such as rhizotrons used for screening roots in soil-free transparent media or in soil, provides a non-destructive way to measure different RSA features under abiotic stressors (Luo et al., 2019; Reni et al., 2025). Other HTP platforms employed for RSA include normalized difference vegetation index (NDVI) and canopy temperature (CT), which help create unbiased models for yield prediction and serve as efficient indirect methods for deep root screening (**Figure 3**) (Li et al., 2019). Therefore, current research should focus on integrating omics approaches for improving RSA in legumes, aiming to develop climate-resilient crop varieties.

## 5 Conclusion and future perspectives

Sustainable crop production and food security are increasingly threatened by unpredictable climate change and severe drought stress. Using OMICS technologies to screen germplasm tolerant to drought stress can potentially speed up genetic improvements in crops under stressful conditions. Functional characterization and cloning of genes identified through OMICS approaches help in understanding both physiological and molecular mechanisms that enable adaptive RSA to mitigate drought stress in legumes. The background and findings presented in this compilation will assist in developing strategies for fine mapping stress-tolerant genes essential for RSA modifications. Likewise, the high-throughput phenotyping of RSA primarily focuses on failed bases results of legume plants tolerant to drought stress (Verheyen et al., 2024). Apart from wonderful applications of OMICS and genetic engineering approaches, recently genome editing technologies assisted by CRISPR/Cas9 (clustered regularly interspaced short palindromic repeat/CRISPR-associated protein 9) has great potential to modulate RSA in plants to alleviate stress conditions (Tariq et al., 2023; Yaqoob et al., 2023; Gao et al., 2025; Alex and Augustine, 2025). The genome editing technologies can modify RSA related alleles to enhance the performance of legume crops for elevated resistance to drought stress.

Artificial intelligence (AI) and Systems biology genetic approach is must for understanding the complex traits and their regulation. Roots, although important for plant development, have been studied very little as such the regulatory network of complex traits is not well understood. For any complex traits, we must have whole system of information. Since roots play important role in water stress and micronutrient uptake, we believe that research on root-omics is need of hours. It is more important when we talk about legumes, as they have more important role to play due to presence of nodules and nutrient assimilation capacity. This review gives overall insights about research done in legume roots and the importance of root studies in understanding system genetics of crop plants for sustainability.
